# His bundle pacing therapy for patients with chronic heart failure: A protocol for meta-analysis based on prospective studies

**DOI:** 10.1097/MD.0000000000025079

**Published:** 2021-03-26

**Authors:** Yongzheng Zhang, Mingwang Ding, Zhihong Pu, Wenjia Peng

**Affiliations:** Department of CCU, Gansu Provincial Third Hospital, Lanzhou, China.

**Keywords:** chronic heart failure, his bundle pacing, meta-analysis, prospective studies

## Abstract

**Introduction:**

A considerable amount of studies have been published with the results of clinical benefit from his bundle pacing (HBP) for chronic heart failure (CHF) patients and these researches led a uncertain conclusion for clinics. Thus, we will conduct a meta-analysis to evaluate the effect of his bundle pacing therapy for chronic heart failure.

**Methods:**

Pubmed, EMBASE, the Cochrane Library, Web of Science and Chinese online databases including Chinese National Knowledge Infrastructure, China Biology Medicine disc, Chinese Scientifific Journals Database (VIP), and Wanfang Database will be searched from these databases construction to the end of November, 2020. The STATA 13.0 will be used for data synthesis and meta-analysis.

**Results:**

The outcome measures included QRS duration, left ventricular ejection fraction, pacing threshold, New York Heart Association (NYHA), left ventricular end-diastolic diameter, left ventricular end-systolic diameter, mitral regurgitation, tricuspid regurgitation, and BNP will be analyzed and synthesized.

**Conclusion:**

This meta-analysis will evaluate the effect of his bundle pacing therapy for chronic heart failure.

**Registration number:**

INPLASY202110109.

## 1 Introduction

Chronic heart failure (CHF), one of the most common causes of hospitalization, is a syndrome characterized by a social impact and clinical significant for both high mortality and morbidity.^[1]^ In recent decades, almost 190,000 hospital admissions for heart failure occurred in patients older than 65 years in US.^[2]^ Cardiac resynchronization therapy has been used for patients with heart failure, reduced left ventricular ejection fraction, and a wide duration of QRS.^[3]^ However, either cardiac resynchronization therapy or biventricular pacing did not seem to derive a better benefit for CHF patients. His bundle pacing (HBP) has recently emerged as an option to deliver physiological ventricular pacing.^[4]^ A considerable amount of studies have been published with the results of clinical benefit from HBP for CHF patients and these researches led a uncertain conclusion for clinics.^[5]^ Thus, we will conduct a meta-analysis to evaluate the effect of His bundle pacing therapy for chronic heart failure.

## 2 Methods

### 2.1 Protocol registration

This protocol for meta-analysis was registered on INPLASY website (https://inplasy.com/inplasy-2021-1-0109/), and INPLASY registration number is INPLASY202110109 (DOI:10.37766/inplasy2021.1.0109). The procedure of this protocol will be conducted according to the guideline of Preferred Reporting Items for Systematic Review and Meta-Analysis Protocols.^[6]^

### 2.2 Search strategy

Pubmed, EMBASE, the Cochrane Library, Web of Science and Chinese online databases including Chinese National Knowledge Infrastructure, China Biology Medicine disc, Chinese Scientifific Journals Database (VIP), and Wanfang Database will be searched from these databases construction to the end of November, 2020. A combination of free term and subject term will be used, and search language was limited to English and Chinese. The key words include: his bundle pacing, HBP, chronic heart failure, CHF, prospective study, randomized controlled trails, RCT.

### 2.3 Inclusion criteria

(1) The subjects were heart failure patients over 18 years old.
  The patient's nationality, race are not limited in this study.
(2) The intervention was his bundle pacing.
(3) The outcome measures included: 1) QRS duration; 2) left ventricular ejection fraction; 3) pacing threshold; 4) New York Heart Association (NYHA) classification of cardiac function; 5) left ventricular end-diastolic diameter; 6) left ventricular end -systolic diameter; 7)mitral regurgitation; 8) tricuspid regurgitation; 9)B-type natriuretic peptide (BNP). Among them, 1) and 2) were the primary outcomes, and the rest were secondary outcomes.
(4) Research types include prospective study, randomized controlled trails. The language of the article is Chinese and English.

### 2.4 Studies selection

**Figure 1:**
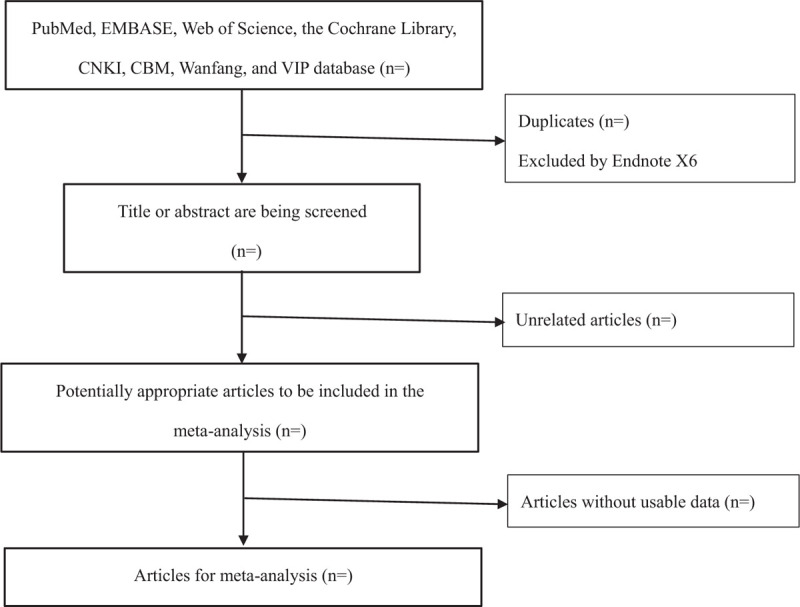
The flowchart of study selection process.

Two researchers (YZZ, MWD) independently screened the literatures according to the search strategy. In the process of screening, researchers need to strictly screen literatures according to the inclusion and exclusion criteria. The 2 researchers carefully read the titles and abstracts of the preliminary literatures, excluded the clinical studies that obviously did not meet the inclusion criteria, and then carefully read the full text of the remaining literatures, considering the research objects, intervention measures, trial design methods and observation outcome indicators of these literatures, so as to determine whether the research can be included. After that, the 2 researchers cross checked the included studies. When they had different opinions, the third researcher assisted to decide whether to include the studies after carefully reading the literature. The progress of studies selection was showed in Figure 1.

### 2.5 Data extraction

Two researchers (YZZ and WJP) extracted the required data according to the designed data extraction table, including:

1. general data of the included studies including the title, authors, the year of publication, type of design;
2. the characteristics of the included studies, such as sample size, gender composition, average age, duration of follow-up, number of deaths, incidence of complications, etc;
3. specific values of the observed outcome indicators at baseline and follow-up.

### 2.6 Assessment of risk of bias in included studies

Two researchers independently evaluated the quality of the included studies. The evaluation tool was the Newcastle-Ottawa scale (NOS). When the total score of NOS in the literature evaluation was ≥7, the study was of regarded as high quality. When they have different opinions, the third researcher will assist. After carefully reading the literature, the total score of NOS in the literature will be determined.

### 2.7 Data synthesis

The STATA 13.0 will be used for data synthesis and meta-analysis. For bivariate data, we use the effect scale index and relative risk ratio of 95% confidence interval (95% CI), whereas the continuous data are represented by mean difference or standardized mean difference and 95% CI. The 95% CI depends on whether the measurement scale is consistent or not. When *P* < .01, the data are considered to be statistically significant. χ^2^ test and *I*^2^ test are used to determine whether there is heterogeneity. If *I*^2^ < 50%, *P* > .1, we can think that there is no heterogeneity in the data analysis, then choose the fixed effect model comprehensive data. If *I*^2^ > 50%, *P* < .1, indicating that there is statistical heterogeneity, the random-effect model is used for analysis. Finally, the subgroup analysis was carried out according to the different causes of heterogeneity. If meta-analysis cannot be performed, a general descriptive analysis can be taken. If the results are heterogeneous, we will take a subgroup analysis of possible factors that may lead to heterogeneity, such as the sex, age, race, BNP level, NYHA. Sensitivity analysis can be carried out when the subgroup analysis is not satisfactory, and it is mainly used to evaluate the robustness of the main results.

## 3 Discussion

The aim of this meta-analysis is to evaluate the efficacy of HBP in patients with CHF. QRS duration represents the time of ventricular electrical activity.^[7]^ The deterioration of uniformity of ventricular electrical activity and the increase of myocardial electrical instability can lead to the prolongation of QRS duration.^[8]^ The influencing factors include the use of class I antiarrhythmic drugs, electrolyte disturbance, myocardial dilation and fibrosis, myocardial ischemia, cardiac dysfunction.^[9]^ The QRS duration can provide a certain reference basis for the evaluation and prediction of clinical efficacy.^[10]^ Its prolongation is one of the possible risk factors of cardiac dysfunction and HF in patients, and can be used as a predictor of cardiac dysfunction after pacing.^[11]^

In addition to echocardiographyd, BNP and NYHA cardiac function classification have their own important clinical significance in the diagnosis and prognosis of HF.^[12]^ Among them, NYHA cardiac function classification is a kind of artificial evaluation and judgment of cardiac function by clinicians according to patients’ chief complaint and self feeling activity ability. It is limited and influenced by many factors, such as patients’ self feeling judgment, patients’ expression and understanding ability, clinicians’ inquiry skills and judgment ability, which can’t reflect or even represent the state of CHF.^[13]^ BNP is a hematological index that can reflect the changes of cardiac function caused by changes in cardiac structure, which is more sensitive to the diagnosis of HF, the judgment of the risk degree and prognosis of patients with HF, and has great guiding significance for clinical practice.^[14]^ Studies have confirmed that the serum BNP level of HF patients is closely related to NYHA cardiac function classification, and left ventricular ejection fraction .^[15]^ It is an important index for clinical diagnosis of HF, evaluation of treatment effect and pre judgment, and can reflect the cardiac function from different sides.^[16]^

In summary, we drafted this scheme to analyze and summarize the efficacy and safety of effective components of HBP in the treatment of CHF. This scheme will conduct meta-analysis for the existing clinical literature, and provide clear evidence-based medicine in the treatment of CHF; it can better for clinical treatment.

## Author contributions

**Conceptualization:** Yongzheng Zhang, Zhihong Pu.

**Data curation:** Yongzheng Zhang, Mingwang Ding, Zhihong Pu.

**Formal analysis:** Yongzheng Zhang.

**Investigation:** Mingwang Ding, Zhihong Pu.

**Methodology:** Zhihong Pu, Wenjia Peng.

**Project administration:** Yongzheng Zhang, Mingwang Ding, Wenjia Peng.

**Resources:** Mingwang Ding, Zhihong Pu, Wenjia Peng.

**Software:** Mingwang Ding.

**Supervision:** Yongzheng Zhang.

**Validation:** Mingwang Ding, Zhihong Pu.

**Visualization:** Mingwang Ding, Wenjia Peng.

**Writing – original draft:** Yongzheng Zhang, Zhihong Pu, Wenjia Peng.

**Writing – review & editing:** Yongzheng Zhang, Zhihong Pu.
